# Hot Tensile Deformation Mechanism and Fracture Behavior of the ZW31/PMMC Laminate

**DOI:** 10.3390/ma16237446

**Published:** 2023-11-30

**Authors:** Dingge Fan, Cuiju Wang, Xuanchang Zhang, Kaibo Nie, Kunkun Deng

**Affiliations:** 1 Shanxi Key Laboratory of Advanced Magnesium-Based Materials, College of Materials Science and Engineering, Taiyuan University of Technology, Taiyuan 030024, China; dinggefan1@163.com (D.F.); 21b909039@stu.hit.edu.cn (X.Z.); niekaibo@tyut.edu.cn (K.N.); 2 School of Materials Science and Engineering, Harbin Institute of Technology, Harbin 150001, China

**Keywords:** laminate, hot deformation mechanism, hot tensile fracture mechanism

## Abstract

In this work, a Mg-Zn-Y (ZW31) alloy with good plasticity was introduced into 10 μm 10 vol% SiC_p_/AZ91 composite materials (PMMCs) via the extrusion compound method, and then the ZW31/PMMC laminate was prepared via multi-pass hot rolling. The hot deformation mechanism and elevated temperature tensile fracture mechanism of ZW31/PMMC laminates were studied using the elevated temperature tensile test. The elevated temperature deformation mechanism is influenced by the strain rate. At low strain rates, grain boundary slip is the primary elevated temperature deformation mechanism of the ZW31/PMMC laminate. However, at high strain rates, the activation of pipeline diffusion is facilitated by the particle deformation zone (PDZ) in the PMMC layer with a high dislocation density, leading to the dominance of dislocation climbing as the main mechanism for elevated temperature deformation of the laminate. Additionally, the implementation of a ZW31/PMMC laminate structure effectively inhibits the initiation and propagation of cavities and microcracks within the laminate layer along the normal direction (ND) while simultaneously blunting crack tips via lattice dislocation emission toward the ZW31 layer. Upon cracking of the PMMC layer, stress concentration occurs in the fracture area of the ZW31 layer, ultimately resulting in necking-induced detachment.

## 1. Introduction

The current utilization of magnesium alloy in industry is limited due to its relatively low strength and modulus despite it being the lightest available metal material [1,2]. The addition of silicon carbide particles (SiC_p_) to a reinforced magnesium matrix composite (PMMC) fabricated using the semi-solid stirring technique [3] can substantially improve both the modulus and strength. However, this enhancement typically comes at the expense of plasticity [4,5,6,7]. Recent research findings suggest that manipulating the microstructure and component distribution of graded materials and layered structures can achieve simultaneous improvement in strength and toughness [8]. By utilizing the extrusion composite method, the Mg-Zn-Y (ZW31) alloy, renowned for its exceptional properties, significantly enhances the plasticity of ZW31/PMMC laminate obtained after rolling while maintaining the superior strength of PMMCs [8]. The incorporation of a layered material’s strength is achieved by redistributing strain and generating back stress via a significant strain gradient at the interface [9,10]. The ZW31/PMMC laminate exhibits immense potential in aerospace, automotive manufacturing, and electronic device applications [11,12].

Due to the unique close-packed hexagonal structure of magnesium alloy, the plastic-forming ability of ZW31/PMMC laminates is limited at room temperature. Therefore, it is crucial to investigate its high-temperature performance. The tensile properties of magnesium alloys and their composites at elevated temperatures have been extensively examined via a multitude of studies. The rolled magnesium alloy sheets, such as AZ91 [13], AZ31 [14], and ZK60 [15], typically exhibit superplasticity at elevated temperatures due to their fine grain sizes. The primary deformation mechanism at elevated temperatures is grain boundary slip. The incorporation of SiC_p_ in magnesium alloy results in an increase in dislocation density, thereby influencing the diffusion of atoms and vacancies during thermal deformation and altering the deformation mechanism of magnesium alloy [16,17,18]. The SiC_p_/Mg-5Al-2Ca composite and Mg-5Al-2Ca alloy were subjected to elevated temperature stretching by Zhang et al. [19]. The presence of SiC_p_ was observed to induce localized stress–strain redistribution in the magnesium alloy, thereby impeding grain boundary slip and promoting the mechanism of dislocation climbing. The elevated temperature deformation mechanism of the ZW31/PMMC laminate, considering the simultaneous introduction of SiC_p_ and interface structure, exhibits a significantly more intricate nature compared to that of a single material. Hence, it is imperative to conduct comprehensive research on its behavior under elevated temperatures.

The deformation of magnesium matrix composites at elevated temperatures is prone to the occurrence of defects such as strain concentration, voids, and cracks [20]. However, the incorporation of a laminate interface structure can effectively enhance its formability [8]. The Cu/Al8011/Al5052 laminate, as prepared by Ebrahimi et al. [21], exhibits a layered structure that enhances its work-hardening capability during elevated temperature tensile deformation, resulting in multiple deflections in the growth path of microcracks. Huang et al. [22] also proposed that the interface structure of the Ti-Al composite plate improves the strain balance of the composite plate while simultaneously resulting in an accumulation of internal stress at the interface. Therefore, the interface structure of the ZW31/PMMC laminate significantly influences the local strain evolution and crack distribution of both the ZW31 layer and PMMC layer during elevated temperature tensile processes. It is necessary to further study the fracture behavior of the ZW31/PMMC laminate during elevated temperature tensile testing.

The present study fabricated a ZW31/PMMC laminate via extrusion and multi-pass hot rolling, with the aim of investigating the thermal deformation mechanism in the temperature range of 573–673 K by calculating the constitutive equation for thermal deformation. Additionally, particular emphasis was placed on examining the fracture mechanism under elevated temperature deformation and elucidating the influence of introducing a laminate interface.

## 2. Materials and Methods

### 2.1. Composite Materials

The Mg-Zn-Y(ZW31) alloy and 10 μm 10 vol% SiC_p_/AZ91 composites (PMMCs) were prepared via semi-solid stirring casting in this study. The pure Mg, pure Zn, and Mg-30% Y intermediate alloys were melted under the protection of SF_6_ and CO_2_ gases. The molten metal solution was poured into a preheated steel mold at 200 °C to produce a ZW31 ingot. The preparation method of the composite material was identical to that of the ZW31 alloy. AZ91 alloy was melted and cooled to the semi-solid temperature range, then agitated to induce turbulent flow on the melt surface, followed by the addition of preheated SiC_p_ at 600 °C. After a specified duration of agitation, the furnace temperature was raised to 700 °C for 5 min. Subsequently, upon cessation of the stirring process, the molten metal solution was poured into a preheated mold at 400 °C for die casting in order to obtain PMMC ingots. Finally, the ZW31 alloy and PMMCs underwent a homogenization treatment followed by water quenching. The homogenization processes consisted of two steps for ZW31 (320 °C for 8 h, followed by 430 °C for 16 h) and two steps for PMMCs (380 °C for 2 h, followed by 415 °C for 22 h), respectively.

### 2.2. Preparation of ZW31/PMMC Laminate

The ZW31 alloy and PMMCs underwent heat treatment prior to being sectioned into rectangular samples measuring 40 × 25 × 6 mm and 40 × 25 × 3.5 mm, respectively. Subsequently, a co-extrusion process was employed with an extrusion ratio of 21:1 and an extrusion speed of 0.1 mm/s to fabricate a ZW31/PMMC laminate with dimensions of 20 mm in width and 3.15 mm in thickness. The ZW31/PMMC laminate underwent preheating in an annealing furnace at a temperature of 350 °C for a period lasting 20 min. Subsequently, it was rolled using a roll diameter measuring 130 mm and rotating at a speed of 20 rpm. The initial roll thickness was reduced by 10%, and each subsequent roll further reduced the thickness by 15%. After undergoing 8 rolling passes, the ZW31/PMMC laminate with a final thickness of 1 mm was obtained.

### 2.3. Elevated Temperature Tensile Test

The high-temperature tensile test of the ZW31/PMMC laminate was conducted using an MTS (E45.105) testing machine. The load accuracy is within a ±0.5% deviation from the displayed value. The temperatures tested were 573 K, 623 K, and 673 K, while the strain rates applied were 1.67 × 10^−2^ s^−1^, 8.3 × 10^−3^ s^−1^, and 8.3 × 10^−4^ s^−1^, respectively. Prior to stretching, the sample was preheated for a duration of 10 min and subsequently quenched in alcohol after experimentation to preserve its deformed microstructure.

### 2.4. Material Characterization

The microstructure analysis of the ZW31/PMMC laminate was conducted using a 4XC optical microscope (OM). After undergoing surface roughening and polishing procedures, the optical characteristics of the microstructure were observed. The caustic solution employed in this study consisted of a 4% concentration of oxalic acid (4 g oxalic acid +100 mL distilled water) and a 3.5% concentration of nitrate alcohol (3.5 mL nitric acid + 100 mL alcohol). The metallographic images collected were subjected to analysis using the Image Pro-Plus software, enabling quantification of the average grain size within the ZW31 layer. The statistical accuracy was ensured by analyzing a minimum of three images for each structure. Additionally, the microstructure and tensile fracture of the ZW31/PMMC laminate were examined using MIRA 3XMU scanning electron microscopy (SEM), while energy dispersive spectroscopy (EDS) was employed for elemental analysis of its structure.

## 3. Results

### 3.1. The Flow Stress Curves

**Figure 1:**
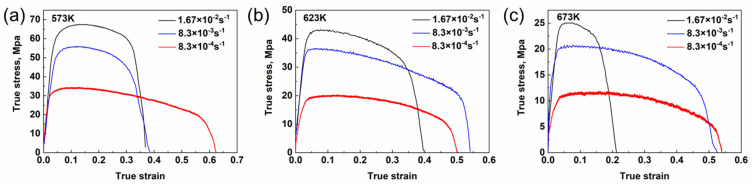
True strain−true stress curves of the as-rolled ZW31/PMMC laminate at different temperatures: (**a**) 573 K, (**b**) 623 K, and (**c**) 673 K.

The stress–strain curves of the ZW31/PMMC laminate, obtained from high-temperature tensile testing under various deformation conditions, are depicted in Figure 1a–c. The elevated temperature tensile stress–strain curve can generally be divided into four distinct stages: elastic deformation, uniform plastic deformation, diffusion necking, and local necking [23,24,25]. During the strain hardening stage, the flow stress undergoes a significant increase due to the proliferation and accumulation of dislocations, leading to an augmented resistance toward deformation. Furthermore, dynamic recrystallization (DRX) serves as the primary mechanism for softening magnesium alloy due to its low fault energy. The nucleation and growth of DRX are accompanied by dislocation annihilation. After reaching the critical DRX strain, the softening mechanism of dislocation annihilation decelerates the increase in flow stress. Subsequently, upon reaching peak stress, diffusion necking occurs, revealing micro-defects such as cavities and microcracks. Moreover, the necking zone exhibits a significant enhancement in strain hardening and deformation resistance, which subsequently propagates to the more susceptible section of the material. The expansion and growth of cavities or microcracks within the specimen ultimately halt necking diffusion, leading to eventual fracture in regions experiencing severe localized necking. During elevated temperature tensile testing, the deformation process observed in ZW31/PMMC laminates is characterized by a competitive interplay between strain hardening, dynamic softening, and cavity/microcrack formation [25,26].

### 3.2. Constitutive Models

The constitutive equation is modeled by the Arrhenius-type function and the Zener–Hollomon parameter (*Z*), which are given by the following equations [27]:

(1) $$ἐ=\{\begin{array}{ll} A_{1}\sigma^{n_{1}}\;\;\exp(-\frac{Q}{RT}) & \;\;\;\;\alpha \sigma <0.8\;\;\;\;\\ A_{2}\exp(\beta \sigma )\exp(-\frac{Q}{RT}) & \;\;\;\;\alpha \sigma >1.2\;\;\;\;\;\\ A{\left[sinh\left(\alpha \sigma \right)\right]}^{n}exp(-\frac{Q}{RT}) & \;\;\;for\;all\;\sigma \;\;\;\; \end{array}$$

(2) $$Z=ἐexp\left(\frac{Q}{RT}\right)$$

where *ἐ* is the strain rate (s^−1^); *R* is 8.314 J/(mol×K); *Q* is the hot deformation activation energy (J/mol); σ is the flow stress (MPa); here, stress is peak stress; *α* and *β* are the stress multiplier; α (=β/n_1_), *A*_1_, *A*_2_, and *A* are material parameters; *n* and *n*_1_ are the stress exponents; Based on Equation (1), the hot deformation activation energy is shown as follows:

(3) $$Q=R{\left(\frac{\partial \ln\left(\sigma \right)}{\partial \left(\frac{1}{T}\right)}\right)}_{ἐ}{\left(\frac{\partial \ln ἐ}{\partial \ln\left(\sigma \right)}\right)}_{T}$$

(4) $$Q=R{\left(\frac{\partial \sigma }{\partial \left(\frac{1}{T}\right)}\right)}_{ἐ}{\left(\frac{\partial lnἐ}{\partial \sigma }\right)}_{T}$$

(5) $$Q=R{\left(\frac{\partial ln\left(sinh\left(\alpha \sigma \right)\right)}{\partial \left(\frac{1}{T}\right)}\right)}_{ἐ}{\left(\frac{\partial lnἐ}{\partial ln\left(sinh\left(\alpha \sigma \right)\right)}\right)}_{T}$$

**Figure 2:**
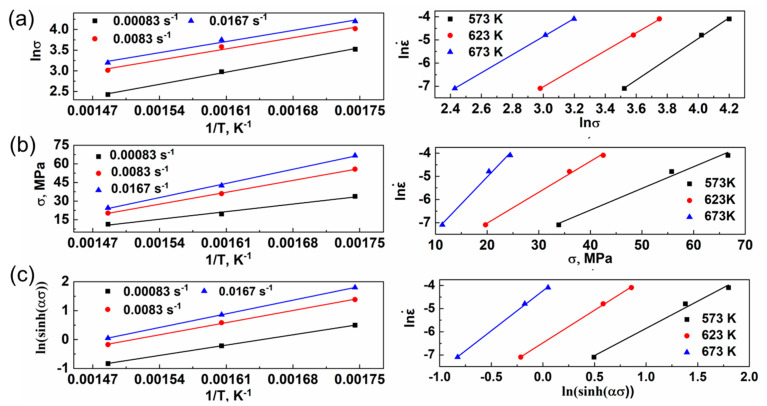
Relationships of (**a**) lnσ − 1/T and lnἐ − lnσ, (**b**) σ − 1/T and lnἐ − σ, and (**c**) ln (sinh(ασ)) − 1/T and lnἐ − ln(sinh(ασ)) for ZW31/PMMC laminate.

According to Equation (3), the *Q* value can be obtained via the mean slope of the lnἐ − lnσ and lnσ − 1/T curve, as shown in Figure 2a,b. Meanwhile, the *Q* values according to Equations (4) and (5) can be calculated using Figure 2c. Substitute Equation (1) into Equation (2) and take the logarithms on both sides:

(6) $$lnZ=n_{1}ln\sigma +lnA_{1}$$

(7) $$lnZ=\beta \sigma +lnA_{2}\;$$

(8) lnZ=nln(sinh(ασ))+lnA

**Figure 3:**
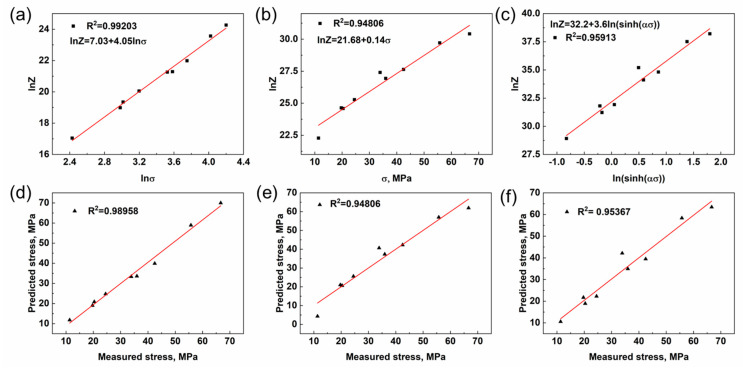
(**a**–**c**) Relationships between lnZ − lnσ, lnZ − σ, and lnZ − ln (sinh (ασ)). (**d**–**f**) Relationships between the predicted stress and measured stress.

The respective relationships between ln*Z* − ln*σ*, ln*Z* − *σ*, and ln*Z* − ln(sinh(ασ)) are illustrated in Figure 3a–c, while Equations (6)–(8) represent the linear relationship. The slope of *n*_1_ is denoted as *β*, and the value of *n* remains unchanged. The linear relationship between the stress values predicted by the three constitutive equations and the experimental values is illustrated in Figure 3d–f. Notably, the power constitutive equation exhibits the highest linear correlation coefficient of 0.98, indicating its exceptional prediction accuracy. Consequently, it serves as a robust theoretical foundation for analyzing the thermal deformation behavior of the ZW31/PMMC laminate.

(9) $$ἐ=1137.76\sigma^{4.05}exp\left(-\frac{135037}{8.314T}\right)$$

The ZW31/PMMC laminate exhibits an activation energy (*Q*) value of 135 kJ/mol and a corresponding *n* value of 4.05. The stress index *n* can generally predict various deformation mechanisms: when *n* = 1, it corresponds to diffusion creep; when *n* = 2, it represents the grain boundary slip (GBS) mechanism; for *n* = 3, it signifies the viscous dislocation slip mechanism; and for *n* = 5–7, it indicates the climbing mechanism of dislocation. Lastly, when *n* = 8, it aligns with the microstructure invariant model mechanism [20]. The stress index of the ZW31/PMMC laminate derived from the power constitutive equation is 4.05, indicating a deviation from established deformation mechanisms. The observed discrepancy can be ascribed to a multitude of factors, including the conditions under which deformation occurs and the structure of layer interfaces. It is worth noting that the mechanism governing material deformation generally exhibits variations in response to changes in temperature or strain rate.

**Figure 4:**
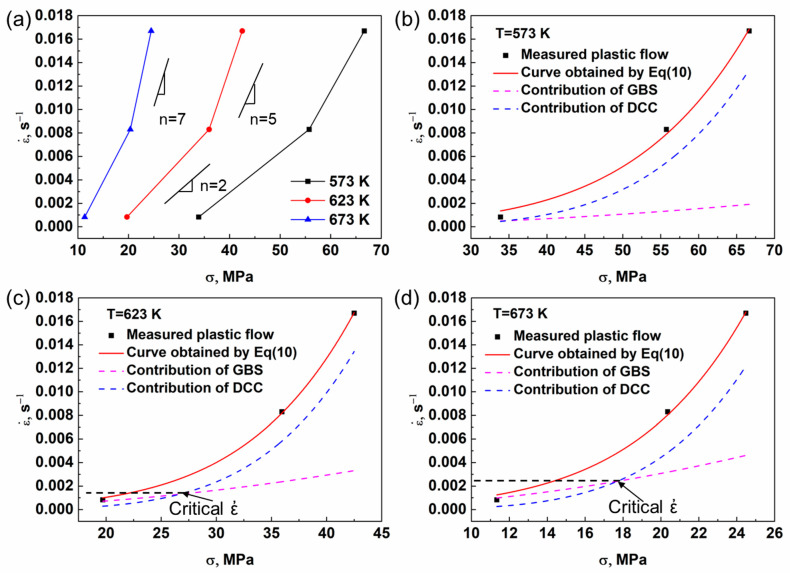
(**a**) Relationship of strain rate and flow stress at various temperatures. (**b**–**d**) Curve fitting of Equation (10).

The value of *n*, representing the slope of strain rate and stress as depicted in Figure 4a, tends to converge toward 2 at lower strain rates. The process of plastic deformation encompasses the simultaneous operation of diverse independent high-temperature deformation mechanisms [28]. The ZW31/PMMC laminate may exhibit distinct elevated temperature deformation mechanisms in the ZW31 layer and the PMMC layer, resulting in a stress index (*n* = 4) that reflects the combination of these different mechanisms. The rolled ZW31/PMMC laminate demonstrates a sub-10 μm grain size, where grain boundary slip (*n* = 2) emerges as the primary high-temperature deformation mechanism in these refined magnesium alloys [29,30]. The incorporation of SiC_p_ into the PMMC layer effectively impedes grain boundary slip, resulting in dislocation climbing becoming the predominant mechanism for elevated temperature deformation. The combined action of grain boundary slip and dislocation climbing is mathematically described by Equation (10) [28].

(10) $$ἐ=B\frac{D_{eff}{}^{*}}{d^{2}}{\left(\frac{\sigma }{E}\right)}^{2}+C\frac{D_{eff}}{b^{2}}{\left(\frac{\sigma }{E}\right)}^{5}$$

(11) $$E=4.3\times 10^{4}\left[1-5.3\times 10^{-4}\left(T-300\right)\right]$$

(12) $$D_{eff}{}^{*}=D_{L}+0.01f_{gb}D_{gb}$$

(13) $$D_{L}=D_{0L}exp\left(-\frac{135000}{RT}\right)$$

(14) $$D_{gb}=D_{0gb}exp\left(-\frac{92000}{RT}\right)$$

(15) $$D_{eff}=D_{L}+50\alpha (\frac{\sigma }{E}{)}^{2}D_{p}$$

(16) $$D_{p}=D_{0p}exp\left(-\frac{92000}{RT}\right)$$

where D_eff_* and *D_eff_* are the effective diffusion coefficients of grain boundary diffusion and dislocation climbing, respectively, *d* is the grain size, *E* is Young’s modulus (MPa), *b* is the Burger vector of Mg alloy (*b* = 3.21 × 10^−10^ m), and *f_gb_* is the fraction of atoms associated with grain boundaries (*f_gb_* = 2bπδ/d). *D_L_*, *D_gb_*, and *D_p_* are the lattice diffusion coefficient, grain boundary diffusion coefficient, and pipe diffusion coefficient of pure magnesium, respectively, where *D_0L_* = 10^−4^ m^2^/s, *D_0gb_* = 7.788 × 10^−3^ m^2^/s, and *D_0p_* = 3.639 × 10^−5^ m^2^/s and *α* is the constant 0.016 for Mg alloy. For uniformity assurance and universal application, the *B* and *C* values are chosen to obtain the best fit to the hyperbolic sine law constitutive equations curves.

The coefficients of the constitutive Equation (10) for the ZW31/PMMC laminate at various temperatures are presented in Table 1. The variations in grain boundary slip mechanism and dislocation climbing mechanism with strain rate at different temperatures are depicted in Figure 4b–d. At lower strain rates, the contribution of the grain boundary slip mechanism is relatively significant; however, as the strain rate increases, the dominance shifts toward the dislocation climbing mechanism as the primary thermal deformation mechanism for the ZW31/PMMC laminate. The critical strain rate (*ἐ_c_*), which marks the transition from grain boundary slip mechanism to dislocation climbing mechanism, increases with rising temperature. The elevated temperature promotes atomic diffusion and grain boundary slip, leading to an increased contribution of the grain boundary slip mechanism in ZW31/PMMC laminates as the temperature rises.

**Figure 5:**
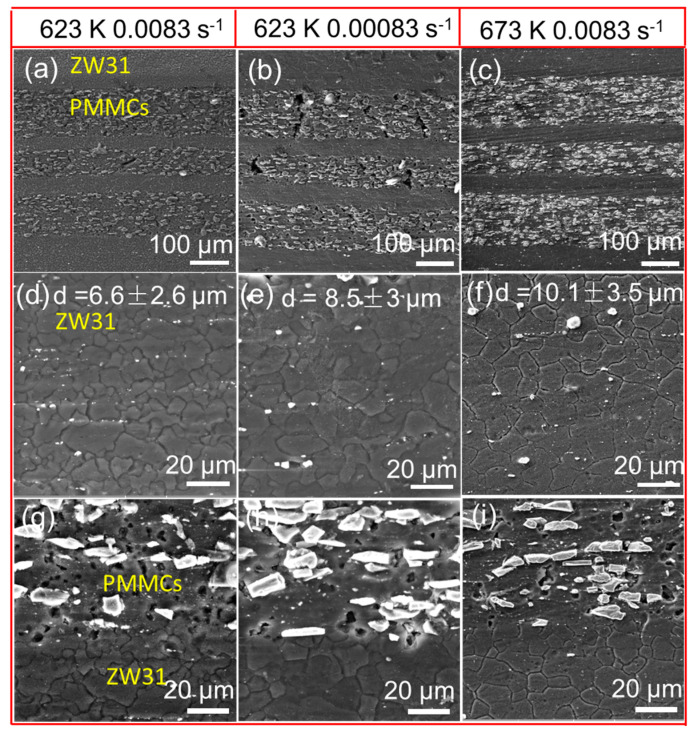
Microstructures of the ZW31/PMMC laminate deformed at (**a**) 623 K, 0.0083 s^−1^, (**b**) 623 K, 0.00083 s^−1^, and (**c**) 673 K, 0.0083 s^−1^, (**d**–**f**) are the microstructures of ZW31 layer under different deformation condition, and (**g**–**i**) are the interface conditions between ZW31 and PMMCs.

The microstructure near the fracture of the sample under various deformation conditions is depicted in Figure 5a–c, while Figure 5d shows that the ZW31 layer has an average grain size of approximately 6.6 μm, indicating a small grain size and an increased number of grain boundaries that provide necessary conditions for the occurrence of grain boundary slip mechanism. With a decrease in strain rate and an increase in temperature, as illustrated in Figure 5e,f, grain growth is observed, which results in average grain sizes of approximately 8.5 μm and 10.1 μm, respectively. The primary mechanism of elevated temperature deformation in the ZW31 layer is attributed to grain boundary slip. The microstructure of the PMMC layer is illustrated in Figure 5g–i, revealing the presence of numerous voids measuring 4–6 μm between SiC_p_ and the matrix. The high-volume fraction of SiC_p_ impedes grain boundary sliding during stretching, resulting in incoherent deformation, stress concentration, and cavitation. A particle deformation zone (PDZ) characterized by a high density of faults and significant orientation differences forms around SiC_p_ during deformation. The elevated dislocation density can facilitate pipe diffusion and enhance the dislocation climbing mechanism [19].

**Figure 6:**
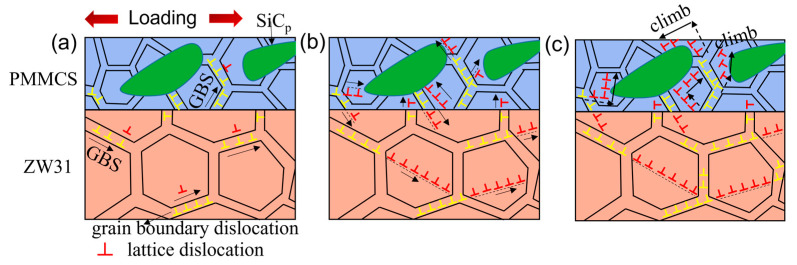
Hot deformation mechanism diagram of the laminate along loading direction at (**a**) low strain rates, (**b**) medium strain rates, and (**c**) high strain rates.

The high-temperature deformation mechanism of the ZW31/PMMC laminate is depicted in Figure 6, showing the grain boundary slip mechanism as the predominant deformation mechanism for both the ZW31 layer and PMMC layer at lower strain rates, as illustrated in Figure 6a [31]. When subjected to shear stress at low strain rates, the adjacent grains in the ZW31 and PMMC layer undergo relative sliding along grain boundaries, resulting in the displacement of grain boundary dislocations. As the strain rate increases, the movement process of grain boundary dislocation is hindered at the triple junction and SiC_p_ within the grain, resulting in hindering grain boundary sliding [32,33,34]. The emission of lattice dislocations via the grain boundary dislocations to adjacent grains at this stage aims to facilitate grain boundary sliding, as illustrated in Figure 6b. The dislocation climb in the PMMC layer can be activated at a high strain rate, as shown in Figure 6c. The stress concentration in the vicinity of SiC_p_ can activate dislocation sources within grains when it reaches the critical shear stress. The particle deformation zone (PDZ) with a high dislocation density stimulates the diffusion of atoms in grains, which facilitates dislocation climb. Zhang et al. [19] demonstrated that the local stress/strain redistribution caused by SiC_p_ can promote dislocation climb creep via finite element simulation.

### 3.3. Fracture Mechanism

**Figure 7:**
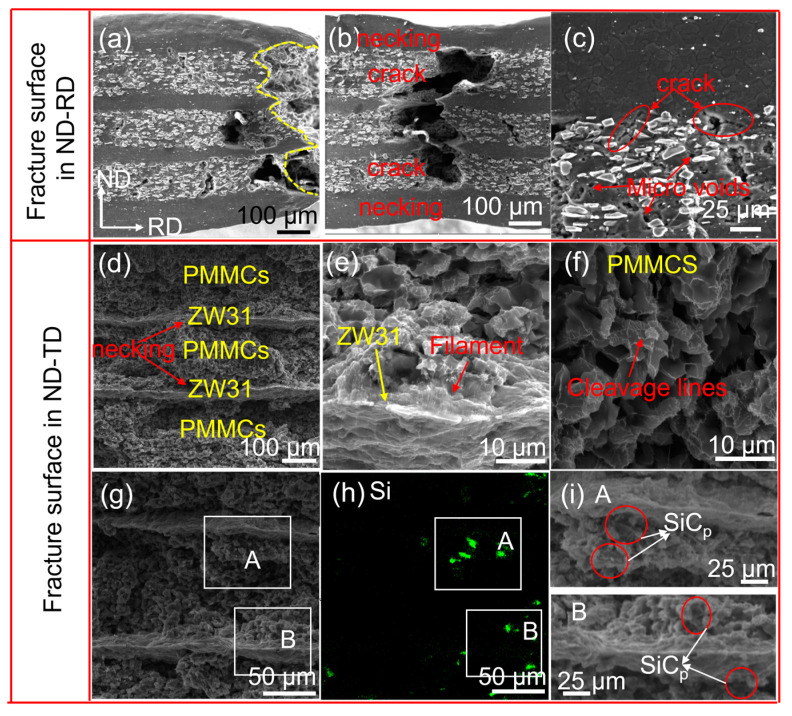
Fracture morphologies of ZW31/PMMC laminate deformed at 623 K and 0.00083 s^−1^. (**a**–**c**) the fracture surface in ND–RD direction; (**d**–**i)** the fracture surface in ND–TD direction.

The front and side fractures of the specimen at a strain rate of 0.00083 s^−1^ and temperature of 573 K are depicted in Figure 7. Notably, the macrofracture on the side of the ZW31/PMMC laminate (Figure 7a) demonstrates an absence of discernible delamination phenomena, thereby indicating a well-adhered interface and effective coordination of deformation across different layers during the deformation process. In Figure 7b, it is evident that the outer ZW31 layer experiences necking subsequent to the fracture of both the inner PMMC layer and the ZW31 layer. Moreover, Figure 7c demonstrates that the microcracks within the PMMC layer terminate at the interface without propagating into the ZW31 layer.

The frontal fracture of the sample reveals distinct fracture characteristics between the ZW31 layer and the PMMC layer, as illustrated in Figure 7d. The presence of SiC_p_ at the fracture site is evident in Figure 7g–i. The high-magnification diagram of the fracture morphology of the PMMC layer, as illustrated in Figure 7f, reveals that PMMCs exhibit intergranular fracture, with the fracture mode being either intergranular cleavage or quasi-cleavage. Under elevated temperature conditions, the constraint of plastic flow near SiC_p_ may lead to the formation of a limited number of voids at the interface between SiC_p_ and matrix. This phenomenon accelerates defect growth at the interface, thereby initiating cracks upon void coalescence. Ultimately, these cracks propagate throughout the PMMC layer, resulting in fracture. Additionally, a prominent conical necking zone is observed within the ZW31 layer. The magnified image of the ZW31 layer is depicted in Figure 7e, revealing distinct tearing edges at the leading fracture and numerous filaments dispersed along the refined grain boundaries. The filaments in the Mg-3Al-Zn alloy were analyzed by Tan et al. [35] using EDS, demonstrating a predominant presence of Zn elements. The formation of filaments is primarily attributed to the diffusion of solute Zn atoms in the neighboring grain boundaries, thereby leading to the generation of voids within the adjoining grain boundaries when these filaments rupture between adjacent grains.

**Figure 8:**
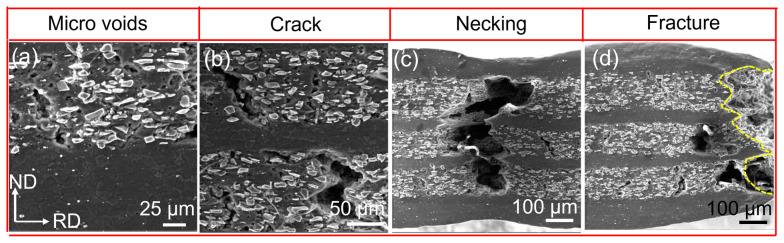
The SEM of fracture process at 623 K and 0.00083 s^−1^: (**a**) microvoids of PMMCs; (**b**) the cracks of PMMCs propagate along ND; (**c**) the necking of the ZW31 layer; and (**d**) the fracture of the ZW31/PMMC laminate.

The fracture mechanism of the ZW31/PMMC laminate was further investigated by conducting SEM observations at various locations on the tensile fracture samples, with a strain rate of 0.00083 s^−1^ and a temperature of 623 K, to examine the initiation and propagation of cracks. The plastic–plastic deformation stage of the ZW31/PMMC laminate is characterized by the initiation of cavities and microcracks within the PMMC layer, followed by their subsequent growth and expansion along the ND direction (Figure 8b). The propagation of cracks is hindered at the interface when microcracks merge to form macroscopic cracks. During the fracture–necking stage, post-necking fracture occurs in the ZW31 layer after the main crack penetrates the PMMC layer, as illustrated in Figure 8c,d.

**Figure 9:**
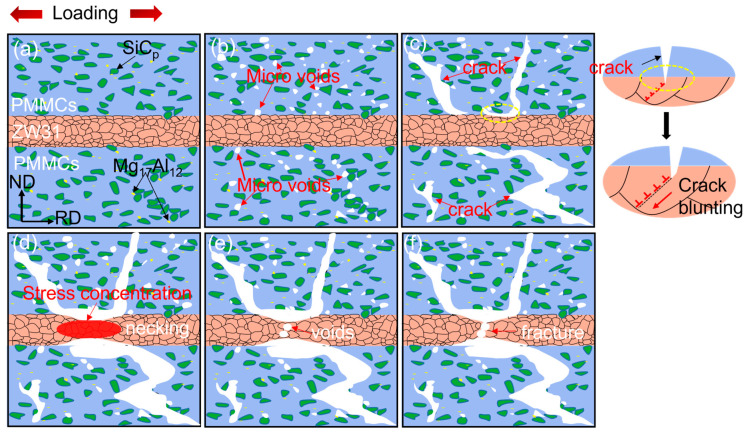
Schematic diagram of the fracture mechanism of ZW31/PMMC laminate under hot deformation. (**a**–**c**) the fracture diagram of the PMMCs layer; (**d**–**f**) the fracture diagram of the ZW31 layer.

The fracture mechanism diagram of the ZW31/PMMC laminate is depicted in Figure 9, based on the aforementioned analysis. The presence of Figure 9b indicates a significant number of vacancies in both SiC_p_ and magnesium matrix, which can be attributed to the weak interface bonding strength between them and hindered grain boundary slip at elevated temperatures. The subsequent aggregation of cavities leads to the initiation and propagation of cracks, primarily propagating along the interface between SiC_p_ and the matrix. Upon reaching the interface between the ZW31 layer and PMMC layer, as shown in Figure 9c, the crack is arrested after propagating along the ND direction. The primary cause of this phenomenon can be attributed to the passivation of the crack tip. In order for crack propagation to occur, it is necessary for the driving force of crack propagation, *J_tip_*, to surpass the resistance against crack propagation [36]. The stress concentration at the crack tip is effectively alleviated by the emission of lattice dislocations [37], as depicted in Figure 9c when the crack propagates into the ZW31 layer of the laminate. This reduction in stress concentration leads to a decrease in *J_tip_* and consequently results in blunting of the crack. Additionally, as illustrated in Figure 9c, during the stage of plastic deformation, a robust synergy between the interface of the PMMC layer and the ZW31 layer induces a triaxial stress state at the crack tip. The ZW31 layer exerts a cohesive force on the PMMC layer along the ND direction, thereby augmenting resistance to crack propagation at the crack tip.

The PMMC layer adjacent to the ZW31 layer is fully fractured as the main crack penetrates it during the progression of plastic deformation. As shown in Figure 9d, the ZW31 layer is subjected to large tensile stress in the fracture zone of the PMMC layer, resulting in stress concentration and a local reduction in the cross-section area (necking) of the ZW31 layer. The neck region of the ZW31 layer undergoes rapid recrystallization and grain boundary migration via diffusion under conditions of elevated temperature and stress. The growth of the cavity is primarily driven by the continuous diffusion of vacancies along the grain boundary, making it challenging for further expansion after nucleation. However, this diffusion process can be impeded by grain boundary migration, leading to creep fracture in the sample akin to “necking” [38], ultimately resulting in the separation of the ZW31 layer.

### 3.4. Effect of the Layer Structure on the Fracture Mechanism

**Figure 10:**
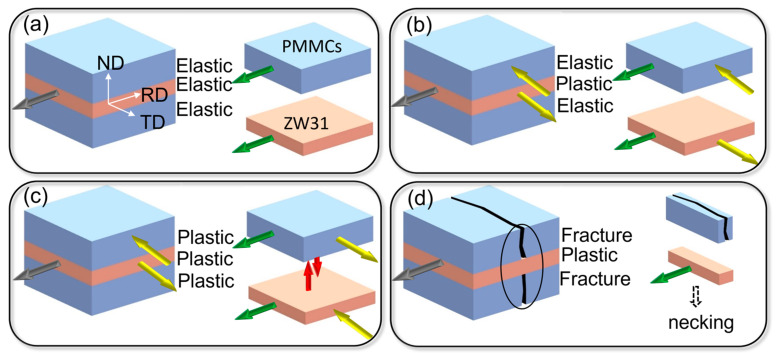
Schematic diagram of the deformation process of ZW31/PMMC laminate. (**a**) the elastic–elastic deformation stage; (**b**) the elastic–plastic deformation stage; (**c**) the plastic–plastic deformation stage; (**d**) is the fracture–plastic deformation stage. The gray arrow represents the stress, and the yellow, green, and red arrows represent the constraining force.

The elevated temperature tensile process for Mg alloy typically consists of four stages: elastic deformation, uniform plastic deformation, diffusion necking, and local necking. A study conducted by Zhang et al. [19] demonstrated that the elevated temperature tensile curve of Mg-5Al-2Ca alloys exhibits a lower strain level at which plastic deformation initiates compared to SiC_p_/Mg-5Al-2Ca composites. The ZW31 layer undergoes primary plastic deformation during the elevated temperature tensile process of the ZW31/PMMC laminate, while the PMMC layer remains in a state of elastic deformation. Once it reaches its yield strength, the PMMC layer also experiences plastic deformation simultaneously with the ZW31 layer. However, due to its low toughness, fracture occurs first in the PMMC layer, leading to stress concentration as the ZW31 layer bears the main tensile stress and subsequently enters the necking stage. The deformation stage of the ZW31/PMMC laminate, as depicted in Figure 10, encompasses the phases of elastic–elastic deformation, elastic–plastic deformation, plastic–plastic deformation, and fracture–necking.

The stress is applied to the ZW31/PMMC laminate along the RD direction, as illustrated in Figure 10a. During the initial stage of elastic deformation, both the ZW31 layer and the PMMC layer deform simultaneously and experience identical strains, thereby satisfying the condition of strain equality. The elastic modulus of the PMMC layer surpasses that of the ZW31 layer, leading to a heightened stress level within the PMMC layer in comparison to the ZW31 layer. The stress zoning behavior becomes apparent at this stage, resulting in the development of internal stress along the layer interface [39]. The ZW31/PMMC laminate exhibits exceptional interfacial bonding, effectively preventing the release of internal stress via interface cracking and instead promoting its accumulation with increasing elastic strain [40].

The incongruent deformation is initiated during the elastic–plastic deformation stage due to the disparity in microstructure and mechanical properties between the ZW31 layer and the PMMC layer. At this stage, as shown in Figure 10b, the ZW31 layer exhibits preferential yielding owing to its lower yield strength, while the PMMC layer continues to undergo elastic deformation. The ZW31 layer experiences tensile stress along the RD direction and undergoes contraction in the TD direction during plastic deformation. However, due to a lack of coordination between the deformation of the ZW31 layer and the PMMC layer, stress is exerted by the PMMC layer on the ZW31 layer along the transverse direction, thereby impeding its deformation along that axis. Due to the variation in strength and flow stress between different layers, a stress gradient is present in the plastic deformation zone, which contributes to an enhancement in the yield strength of the ZW31/PMMC laminate, thereby resulting in synergistic strengthening [41,42].

The plastic deformation stage of the ZW31/PMMC laminate is depicted in Figure 10c. Once the applied stress exceeds the yield strength of the composite material, plastic deformation is initiated within the PMMC layer. The plastic deformation in the PMMC layer, to some extent, enhances the coordination of deformation between the ZW31 layer and the PMMC layer, thereby partially mitigating internal stress along the RD direction. However, due to the limited plasticity of PMMCs, cavities and microcracks tend to develop at the SiC_p_–Mg matrix interface under conditions of low plastic strain. The presence of defects induces rapid shrinkage of the PMMC layer along both the transverse direction (TD) and normal direction (ND). During this process, the lamellar structure acts as a constraint, causing a delay in the contraction of the PMMC layer and resulting in tensile stress exerted by the ZW31 layer along both ND and TD directions.

## 4. Conclusions

(1) The stress index (*n* = 4) of the ZW31/PMMC laminate was determined using the power constitutive equation, which represents a combination of elevated temperature deformation mechanisms controlled by grain boundary slip and dislocation climbing in the ZW31/PMMC laminate.

(2) The deformation mechanism of the ZW31 layer and PMMC layer at elevated temperature is characterized by grain boundary slip at low strain rates, while dislocation climbing becomes dominant at high strain rates due to the presence of PDZ in the PMMC layer with a high dislocation density.

(3) The fracture of the ZW31/PMMC laminate at elevated temperature originates from the PMMC layer and propagates along the ND direction, while the outermost ZW31 layer fractures in a localized necking manner.

(4) The introduction of the ZW31/PMMC laminate layer structure induces internal stress to effectively mitigate plastic deformation in both the alloy and PMMC layers, thereby enhancing the ductility of the composite material.

## Figures and Tables

**Table 1:** Relevant parameters and calculated results of constitutive equation of Equation (10).

T/K	*D_L_*/ × 10^−17^ m^2^/s	*D_gb_* × 10^−17^ m^2^/s	*D_p_* × 10^−13^ m^2^/s	$D_{eff}{}^{*}$ × 10^−16^ m^2^/s	$D_{eff}$ × 10^−17^ m^2^/s	B × 10^7^	C × 10^9^
573	4.9	3.1	1.5	2.5	4.9	4.3	1.4
623	47.9	15.0	7.0	14.2	47.9	1.6	1.2
673	332.0	56.3	26.3	68.7	332.0	1.3	2.1

## Data Availability

The data presented in this study are available on request from the corresponding author.

## References

[1] Zhou S., Liu T., Tang A., Huang Y., Peng P., Zhang J., Hort N., Willumeit-Römer R., Pan F. (2023). Designing Mg Alloys with High Strength and Ductility by Reducing the Strength Difference between the Basal and Non-Basal Slips. Mater. Des..

[2] Wang X.J., Xu D.K., Wu R.Z., Chen X.B., Peng Q.M., Jin L., Xin Y.C., Zhang Z.Q., Liu Y., Chen X.H. (2018). What Is Going on in Magnesium Alloys?. J. Mater. Sci. Technol..

[3] Deng K.K., Shi J., Wang C.J., Wang X.J., Wu Y.C., Nie K.B., Wu K. (2012). Microstructure and Strengthening Mechanism of Bimodal Size Particle Reinforced Magnesium Matrix Composite. Compos. Part Appl. Sci. Manuf..

[4] Kou H.N., Lu J., Li N. (2014). High-Strength and High-Ductility Nanostructured and Amorphous Metallic Materials. Adv. Mater..

[5] Lu K., Lu L., Suresh S. (2009). Strengthening Materials by Engineering Coherent Internal Boundaries at the Nanoscale. Science.

[6] Liu L., Miyake M., Li L., Jia W., Shibutani Y. (2023). Numerical Study on the Ductility Improvement Mechanism of Dilute Mg-Ca Alloys. Comput. Mater. Sci..

[7] Ying T., Yu M.D., Chen Y.W., Zhang H., Wang J.Y., Zeng X.Q. (2022). Dominant Deformation Mechanisms in Mg–Zn–Ca Alloy. Acta Metall. Sin. (Engl. Lett.).

[8] Zhang X.C., Wang C.J., Deng K.K., Nie K.B., Gan W.M., Liang W., Wu Y.C. (2019). Fabrication, Microstructure and Mechanical Properties of the as-Rolled ZW31/PMMCs Laminate. Mater. Sci. Eng. A.

[9] Pang J.C., Fan G.H., Cui X.P., Li A.B., Geng L., Zheng Z.Z., Wang Q.W. (2013). Mechanical Properties of Ti–(SiCp/Al) Laminated Composite with Nano-Sized TiAl3 Interfacial Layer Synthesized by Roll Bonding. Mater. Sci. Eng. A.

[10] Chaudhari G.P., Acoff V. (2009). Cold Roll Bonding of Multi-Layered Bi-Metal Laminate Composites. Compos. Sci. Technol..

[11] Nguyen H.N., Nguyen T.Y., Tran K.V., Tran T.T., Nguyen T.T., Phan V.D., Do T.V. (2019). A Finite Element Model for Dynamic Analysis of Triple-Layer Composite Plates with Layers Connected by Shear Connectors Subjected to Moving Load. Materials.

[12] Nguyen H.N., Hong T.T., Vinh P.V., Thom D.V. (2019). An Efficient Beam Element Based on Quasi-3D Theory for Static Bending Analysis of Functionally Graded Beams. Materials.

[13] Wei Y.H., Wang Q.D., Zhu Y.P., Zhou H.T., Ding W.J., Chino Y., Mabuchi M. (2003). Superplasticity and Grain Boundary Sliding in Rolled AZ91 Magnesium Alloy at High Strain Rates. Mater. Sci. Eng. A.

[14] Watanabe H., Tsutsui H., Mukai T., Kunio I., Okanda Y., Kohzu M., Higashi K. (2000). Superplastic Behavior in Commercial Wrought Magnesium Alloys. Mater. Sci. Forum.

[15] Kim W.J., Kim M.J., Wang J.Y. (2009). Superplastic Behavior of a Fine-Grained ZK60 Magnesium Alloy Processed by High-Ratio Differential Speed Rolling. Mater. Sci. Eng. A.

[16] Fan D.G., Deng K.K., Wang C.J., Nie K.B., Shi Q.X., Wu Y.C. (2020). Improved Workability of an Mg-5 Wt.%Zn Alloy by the Addition of Trace SiCp. Mater. Today Commun..

[17] Fan D.G., Deng K.K., Wang C.J., Nie K.B., Shi Q.X., Liang W. (2021). Hot Deformation Behavior and Dynamic Recrystallization Mechanism of an Mg-5wt.%Zn Alloy with Trace SiCp Addition. J. Mater. Res. Technol..

[18] Wang J., Chen Y., Chen Z., Llorca J., Zeng X. (2021). Deformation Mechanisms of Mg-Ca-Zn Alloys Studied by Means of Micropillar Compression Tests. Acta Mater..

[19] Zhang L., Su K., Deng K., Nie K., Wang C., Liang W. (2020). Hot Tensile Behavior and Deformation Mechanism of Mg–5Al–2Ca Alloy Influenced by SiC Particles. Mech. Mater..

[20] Shi D.F., Ma A., Pérez-Prado M.T., Cepeda-Jiménez C.M. (2023). Activation of Second-Order Pyramidal Slip and Other Secondary Mechanisms in Solid Solution Mg-Zn Alloys and Their Effect on Tensile Ductility. Acta Mater..

[21] Ebrahimi M., Liu G., Wang Q., Jiang H., Ding W., Shang Z., Luo L. (2020). Evaluation of Interface Structure and High-Temperature Tensile Behavior in Cu/Al8011/Al5052 Trilayered Composite. Mater. Sci. Eng. A.

[22] Huang M., Xu C., Fan G., Maawad E., Gan W., Geng L., Lin F., Tang G., Wu H., Du Y. (2018). Role of Layered Structure in Ductility Improvement of Layered Ti-Al Metal Composite. Acta Mater..

[23] Hou M., Deng K., Wang C., Nie K., Shi Q. (2022). The Work Hardening and Softening Behaviors of Mg–6Zn-1Gd-0.12Y Alloy Influenced by the VR/VD Ratio. Mater. Sci. Eng. A.

[24] Cao F.R., Xia F., Xue G.Q. (2016). Hot Tensile Deformation Behavior and Microstructural Evolution of a Mg–9.3Li–1.79Al–1.61Zn Alloy. Mater. Des..

[25] Zhou M., Lin Y.C., Deng J., Jiang Y.-Q. (2014). Hot Tensile Deformation Behaviors and Constitutive Model of an Al–Zn–Mg–Cu Alloy. Mater. Des..

[26] Deng J., Lin Y.C., Li S.-S., Chen J., Ding Y. (2013). Hot Tensile Deformation and Fracture Behaviors of AZ31 Magnesium Alloy. Mater. Des..

[27] Deng K., Li J., Xu F., Nie K., Liang W. (2015). Hot Deformation Behavior and Processing Maps of Fine-Grained SiCp/AZ91 Composite. Mater. Des..

[28] Kim W.J., Chung S.W., Chung C.S., Kum D. (2001). Superplasticity in Thin Magnesium Alloy Sheets and Deformation Mechanism Maps for Magnesium Alloys at Elevated Temperatures. Acta Mater..

[29] Watanabe H., Mukai T., Kohzu M., Tanabe S., Higashi K. (1999). Effect of Temperature and Grain Size on the Dominant Diffusion Process for Superplastic Flow in an AZ61 Magnesium Alloy. Acta Mater..

[30] Mabuchi M., Ameyama K., Iwasaki H., Higashi K. (1999). Low Temperature Superplasticity of AZ91 Magnesium Alloy with NonEquilibrium Grain Boundaries. Acta Mater..

[31] Aifantis K.E. (2009). Interfaces in Crystalline Materials. Procedia Eng..

[32] Yu M., Cui Y., Wang J., Chen Y., Ding Z., Ying T., Llorca J., Zeng X. (2023). Critical Resolved Shear Stresses for Slip and Twinning in Mg-Y-Ca Alloys and Their Effect on the Ductility. Int. J. Plast..

[33] Langdon T.G. (2006). Grain Boundary Sliding Revisited: Developments in Sliding over Four Decades. J. Mater. Sci..

[34] Humphreys F.J., Kalu P.N. (1987). Dislocation-Particle Interactions during High Temperature Deformation of Two-Phase Aluminium Alloys. Acta Metall..

[35] Tan J.C., Tan M.J. (2003). Superplasticity and Grain Boundary Sliding Characteristics in Two Stage Deformation of Mg–3Al–1Zn Alloy Sheet. Mater. Sci. Eng. A.

[36] Ojima M., Inoue J., Nambu S., Xu P., Akita K., Suzuki H., Koseki T. (2012). Stress Partitioning Behavior of Multilayered Steels during Tensile Deformation Measured by in Situ Neutron Diffraction. Scr. Mater..

[37] Pineau A., Benzerga A.A., Pardoen T. (2016). Failure of Metals I: Brittle and Ductile Fracture. Acta Mater..

[38] Xu G.S. (2004). Chapter 65—Dislocation Nucleation from Crack Tips And Brittle to Ductile Transitions in Cleavage Fracture. Dislocat. Solids.

[39] Antolovich S.D., Armstrong R.W. (2014). Plastic Strain Localization in Metals: Origins and Consequences. Prog. Mater. Sci..

[40] Sharon E., Gross S.P., Fineberg J. (1996). Energy Dissipation in Dynamic Fracture Available. Phys. Rev. Lett..

[41] Wu X.L., Jiang P., Chen L., Zhang J.F., Yuan F.P., Zhu Y.T. (2014). Synergetic Strengthening by Gradient Structure. Mater. Res. Lett..

[42] Chakravarthy S.S., Curtin W.A. (2011). Stress-Gradient Plasticity. Proc. Natl. Acad. Sci. USA.

